# A 3D bioinspired highly porous polymeric scaffolding system for *in vitro* simulation of pancreatic ductal adenocarcinoma

**DOI:** 10.1039/c8ra02633e

**Published:** 2018-06-07

**Authors:** Stella Totti, Mark C. Allenby, Susana Brito Dos Santos, Athanasios Mantalaris, Eirini G. Velliou

**Affiliations:** Bioprocess and Biochemical Engineering Group (BioProChem), Department of Chemical and Process Engineering, University of Surrey Guildford GU2 7XH UK e.velliou@surrey.ac.uk 0044-(0)-1483686577; Biological Systems Engineering Laboratory (BSEL), Department of Chemical Engineering, Imperial College London London SW7 2AZ UK

## Abstract

Pancreatic ductal adenocarcinoma is an aggressive disease with an extremely low survival rate. This is due to the (i) poor prognosis and (ii) high resistance of the disease to current treatment options. The latter is partly due to the very complex and dense tissue/tumour microenvironment of pancreatic cancer, which contributes to the disease's progression and the inhibition of apoptotic pathways. Over the last years, advances in tissue engineering and the development of three-dimensional (3D) culture systems have shed more light into cancer research by enabling a more realistic recapitulation of the niches and structure of the tumour microenvironment. Herein, for the first time, 3D porous polyurethane scaffolds were fabricated and coated with fibronectin to mimic features of the structure and extracellular matrix present in the pancreatic cancer tumour microenvironment. The developed 3D scaffold could support the proliferation of the pancreatic tumour cells, which was enhanced with the presence of fibronectin, for a month, which is a significantly prolonged *in vitro* culturing duration. Furthermore, *in situ* imaging of cellular and biomarker distribution showed the formation of dense cellular masses, the production of collagen-I by the cells and the formation of environmental stress gradients (*e.g.* HIF-1α) with similar heterogeneity trends to the ones reported in *in vivo* studies. The results obtained in this study suggest that this bioinspired porous polyurethane based scaffold has great potential for *in vitro* high throughput studies of pancreatic cancer including drug and treatment screening.

## Abstract 2

A 3D biomimetic model for *in vitro* studies of pancreatic cancer.

## Graphical abstract

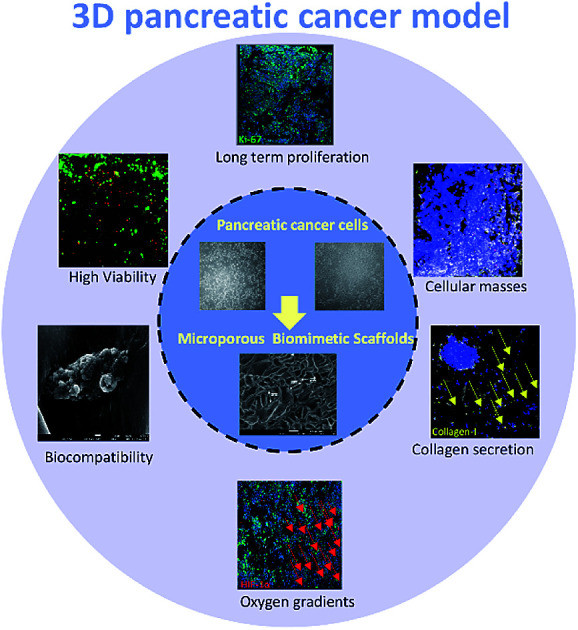

## 1. Introduction

Pancreatic ductal adenocarcinoma (PDAC) is an aggressive malignancy, being the fourth leading cause of cancer deaths in the USA, the fifth in the United Kingdom and the seventh worldwide.^1,2^ The 5 year survival rate, which has barely improved over the last 4 decades, is 8%.^2^ The high disease mortality is due to (i) the lack of symptoms which leads to late stage diagnosis,^3^ (ii) the high metastatic likelihood^4^ and (iii) the high resistance to currently available treatment options.^5^ The latter can be partly attributed to the disease's complex tumour microenvironment (TME),^6–9^ which consists of intense extracellular matrix (ECM) fibrosis (desmoplasia)^6,10–12^ that can contribute to the disease's progression and inhibition of apoptotic pathways, both increasing treatment resistance.^13–15^ Classically used systems for studying pancreatic cancer are either 2D *in vitro* systems or animals. While 2D *in vitro* systems are easy to use and responsive to radiation and drugs^8,16,17^ they cannot accurately simulate important 3D *in vivo* TME aspects such as structure, porosity, presence of ECM proteins, realistic cell–cell and cell–ECM interactions, environmental gradients (nutrient, oxygen) and vascularisation.^8,18–21^ Several animal model systems have been developed for pancreatic cancer,^22,23^ the best of which involve genetic modification, that have provided considerable insights into the pathogenesis of the disease. However, they are time consuming, expensive to generate and maintain and they are not always reproducible.^23–29^ Advances in tissue engineering enable the development of 3D constructs that are generally less expensive than animals, more reproducible and easy to develop while they provide a more realistic structure, cell–cell, cell–ECM spatial interactions and a more realistic distribution of environmental parameters within the system, such as nutrients and oxygen, chemotherapeutic diffusion, and irradiation deposition as compared to 2D systems.^8,30–36^

Current 3D *in vitro* systems of pancreatic tumours include (i) spheroid/organoid systems,^37–45^ (ii) hydrogel scaffolds^42,46–48^ and (iii) polymeric scaffolds.^49–51^ For example, Ware *et al.* have developed pancreatic cancer spheroids with a distinct cohesiveness of the cellular masses.^43^ Additionally, Longati *et al.* showed that pancreatic cancer cell lines in spheroids exhibited decreased cellular proliferation, increased ECM production and resistance to chemotherapeutic reagents as compared to a 2D monolayer culture.^37^ The increased chemo-resistance of pancreatic tumour spheroids in comparison with conventional 2D systems was also reported by Wen *et al.*^44^ Some features of the complex pancreatic cancer TME have been shown within spheroids. For example, Ware *et al.* developed a stroma rich spheroid matrix that exhibited regions of increased collagen expression compared to the non-stromal rich control system.^41^ Furthermore, Boj *et al.* constructed 3D matrigel based organoids of primary PDAC cells in order to model *in vitro* some essential histological and genetic characteristics of this malignancy.^39,40^ Raza *et al.* developed polymeric hydrogels of various stiffness levels. It was observed that the stiffness of the polymeric matrix affected the cellular proliferation.^48^ Similar results were reported by Ki *et al.* who observed increased pancreatic cancer cell growth after 7 days of culture for softer thiol-ene based hydrogels.^47^ Additionally, Chiellini *et al.* created two types of microstructured hydrogels, fabricated either with chitosan (CS) or with a polyelectrolyte complex formed between CS and poly(γ-glutamic acid) (γ-PGA). BxPC-3 pancreatic tumour cells proliferated in those hydrogels for 28 days, they formed aggregates and they retained cancer typical features, *i.e.* loss of polarity and duct-like structures. Additionally, fascin (a marker of tumour invasiveness), was expressed in the hydrogels, but not in 2D monolayer systems.^46^

There are a few limited studies investigating the potential of 3D polymeric scaffolds as a pancreatic cancer model. More specifically, Wang *et al.* showed that a fibrous polyglyconate/gelatine scaffold (4 mm inner diameter) supported the proliferation of pancreatic cancer stem cells (CSCs) *in vitro* for 7 days as well tumour formation and metastasis, when it was transplanted in mice.^50^ Furthermore, He *et al.* created a fibrous polymeric scaffolding system based on poly (glycolide-*co*-trimethylene carbonate) and gelatine for pancreatic tumour growth. This polymeric scaffold model demonstrated better neoplastic formation and accelerated tumour evolution as compared with the 2D system.^51^ Ricci *et al.* developed three different biocompatible scaffolds based on two polymers [poly (ethylene oxide terephthalate)/poly(butylene terephthalate) and poly(vinyl alcohol)/gelatine] and two polymeric formulations (fibre mesh and sponge like). The type of polymer and the formulation technique altered the internal architecture of the scaffold, which affected the cell growth and morphology as well as the tumour-specific metalloproteinases (MMPs) synthesis of PDAC.^49^

Among existing tissue engineering systems, polymeric scaffolds provide a more robust control of the internal structure (pore shape, size and interconnectivity) enabling the TME structural recapitulation.^8,21,30,49,52–56^ Additionally, surface modification of the scaffolds with proteins enables the ECM mimicry which is crucial in tissue and/or tumour development.^21,55,57,58^ Furthermore, the high mechanical strength of polymeric scaffolds allows the introduction of perfusion in the system for vascularization mimicry with tuneable flow rates.^52,54^ Overall, the above features enable the recapitulation of realistic cell–cell interactions and distributions, cell–matrix interactions as well as the formation of realistic oxygen and nutrient gradients. These gradients can be more controlled, tuned and less acute/extreme in highly porous polymeric scaffolds as compared to cell aggregate based 3D systems.^8,35^ Therefore, polymeric scaffolds are a promising platform for pancreatic cancer *in vitro* studies.

Natural and synthetic are the two main categories of polymers that are used for scaffold fabrication in tissue engineering. However, natural polymers are not preferred for load-bearing tissue applications due to their limited mechanical and physical stability and therefore synthetic polymers are currently the dominant scaffolding matrices in tissue engineering and regenerative medicine.^59–61^ Among the synthetic polymers, polyurethanes (PUs) are considered very promising candidates in tissue engineering due to their unique segmented structure, their wide range of mechanical and physical properties, their assortment from stable to degradable materials and their biocompatibility.^61,62^ PUs have been used as scaffolding materials for soft,^63^ cartilage,^64^ bone tissue engineering,^65^ and to recapitulate normal and abnormal haematopoiesis.^21,52,55^ Nevertheless, they have not been used to date for pancreatic cancer tissue engineering.

In this study, we report for the first time the use of a highly porous 3D polyurethane (PU) scaffolding system as a platform for pancreatic cancer modelling. Surface modification of the scaffold took place with fibronectin (FN) for enhancement of cellular adhesion. The cellular evolution including proliferation, morphology and cell mass formation in the scaffold was monitored for 29 days. Furthermore, sectioning of the scaffolds, fluorescent staining and imaging with confocal laser scanning microscopy (CLSM) enabled the spatial 3D determination of environmental (stress) gradients, *i.e.*, oxidative, starvation, and ECM production within the 3D scaffolding system.

## 2. Materials and methods

### 2.1. Fabrication and sterilisation of the 3D scaffolds

**Fig. 1:**
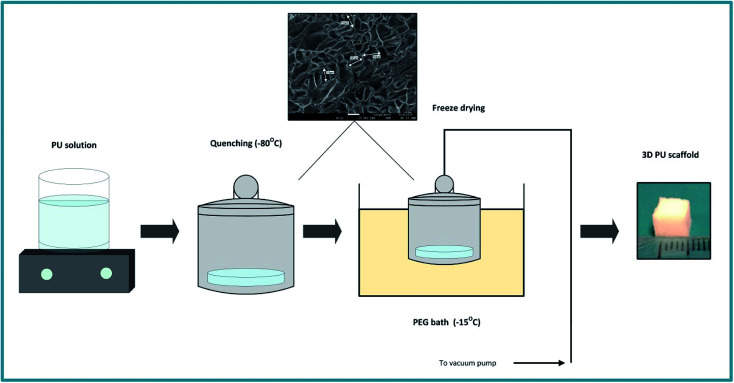
Schematic representation of the PU scaffold fabrication process with the Thermally Induced Phase Separation (TIPS).

The PU scaffolds were fabricated by the Thermally Induced Phase Separation (TIPS) method, which is described in Fig. 1.^21,55,66,67^ PU beads (Noveon, Belgium) were dissolved in dioxan (5% w/v) (99.8% anhydrous pure, Sigma-Aldrich, UK) and the solution was quenched at −80 °C for 2 h. Thereafter, the solvent was removed by freeze drying in a poly-ethylene glycol (PEG) bath at −15 °C under 0.01 mbar vacuum pressure. The scaffolds were then cut in 5 × 5 × 5 mm^3^ cubes. The average pore size was 100–150 μm, the porosity 85–90%, as determined by Mercury Intrusion Porosimetry (MIP) (PoreMaster33, Quantachrome) and the specific pore volume obtained using helium displacement pycnometry (AccuPyc, 1330 V3.00) was 2.3 ± 0.48 cm^3^ g^−1^. The compression modulus was 28 ± 3 kPa, as reported previously by Safinia *et al.*^67^ Sterilisation of the scaffolds took place by washing them with 70% v/v ethanol solution for 3 h followed by exposure to a UV/ozone generator for 10 min (BioForce Nanosciences, USA).

### 2.2. Surface modification of the 3D scaffolds

The PU scaffolds were coated with fibronectin (FN) from bovine plasma (Sigma-Aldrich, UK), to mimic a crucial ECM protein dominant in pancreatic cancer as well as to enhance the cell adhesion on the PU matrix. The protein coating took place *via* adsorption of the FN on the PU.^68,69^ More specifically, as previously described,^21,55^ the scaffolds were dipped in Phosphate-Buffered Saline (PBS, Sigma-Aldrich, UK) for 10 min and centrifuged (in PBS) for 10 min at 2500 rpm. Then, they were transferred to the FN solution and centrifuged for 20 min at 2000 rpm. Centrifuging ensures better penetration and uniform distribution of the protein solution in the PU matrix. Then, one more centrifugation step in PBS for 10 min at 1500 rpm is carried out to unblock the surface pores of the scaffolds. The FN coating concentration was 25 μg mL^−1^.^21,55,70,71^

### 2.3. 3D cell culture

The human pancreatic adenocarcinoma cell lines AsPC-1 (ATCC® UK, CRL-1682) and PANC-1 (Sigma-Aldrich UK, ECAAC 87092802) were expanded in tissue culture plastic flasks (Fisher Scientific, UK) in Dulbecco's modified Eagle's medium (DMEM) with high glucose (Lonza, UK), while the BxPC-3 (ATCC® UK, CRL-1687) cell line was cultured in Roswell Park Memorial Institute (RPMI) 1640 medium (Lonza, UK) with high glucose, according to the supplier's culture protocol, in a humidified incubator at 37 °C and 5% CO_2_ until an adequate number of cells for each experiment was achieved. Both culture media were supplemented with 10% v/v heat inactivated foetal bovine serum (Fisher Scientific, UK), 100 U mL^−1^ penicillin/streptomycin (Sigma-Aldrich, UK) and 2 mM l-glutamine (Sigma-Aldrich, UK) stock solutions. Mycoplasma negative cultures were ensured by regular mycoplasma screening tests (Lonza, UK).

For all conditions under study, 100 μL of cell suspension (5 × 10^5^ cells per 100 μL) was seeded in sterile scaffolds, which were placed in 24 well-plates and allowed to settle for 15 min in an incubator at 37 °C in a 5% CO_2_ atmosphere. Then, 1.5 mL of the appropriate cell culture medium was added in each well. Scaffolds were placed in an incubator at 37 °C, 5% CO_2_ and 20% O_2_. Cell growth was monitored for 29 days (5 weeks). Cell culture medium was replenished every two to three days. In order to avoid cell confluency at the bottom of the wells resulting from scaffold egress, scaffolds were placed in a new well-plate on a weekly basis.

### 2.4. MTS cell viability assay

Quantitative assessment of cell viability/proliferation in the scaffolds took place by measuring the increase of metabolically active cells using the tetrazolium compound [3-(4,5-dimethylthiazol-2-yl)-5-(3-carboxymethoxyphenyl)-2-(4-sulfophenyl)-2*H*-tetrazolium] MTS reagent (Promega, CellTiter96® Aqueous Solution Cell Proliferation Assay, WI USA). The reagent was supplemented to fresh culture medium at a ratio of 1 : 5 according to the manufacturer's protocol and added to the 3D scaffold cultures prior to a 3 h incubation at 37 °C, according to the manufacturer's protocol. Afterwards, the absorbance was measured at 490 nm on a plate reader (Synergy HT, BioTek, VT, USA).

### 2.5. Scanning electron microscopy (SEM)

The 3D scaffolds were sectioned and the cell distribution and adhesion on/in the pores of the matrix was observed with scanning electron microscopy (SEM). More specifically, scaffolds were collected at the beginning (day 1) and at the culture endpoint (day 29), snap frozen in liquid nitrogen for 15 min and then preserved in −80 °C until fixation. At fixation, samples where sectioned approximately in the middle with a razor and then directly immersed in 4% v/v cold formaldehyde solution (Sigma-Aldrich, UK) for 2 h at room temperature. Thereafter, post fixation took place including 2 washing steps with PBS for 15 min each, followed by 4 washing steps with deionized water for 15 min each to ensure removal of the residual crystals. Afterwards, the scaffold sections were air dried overnight in an aseptic environment. The specimens were sputter coated with gold in an argon atmosphere 24 h prior the SEM imaging. The scanning electron microscopy was performed on a JOEL JMS610LA (JEOL USA, MA, USA) microscope at different magnifications.

### 2.6. Immunofluorescence assays

*In situ* immunofluorescence (IF) staining of the scaffolds took place for the spatial determination of the (i) cell organisation and/or cell masses formations (4′,6-diamidino-2-phenylindole, DAPI), (ii) cell proliferation (Ki-67), (iii) ECM production (collagen-I), (iv) potential environmental stress gradients, *i.e.*, oxygen/oxidative stress (HIF-a) and nutrient/starvation stress (LC3). More specifically, scaffolds were collected at the middle (day 15) and the end of the culturing period (day 29), snap frozen in liquid nitrogen for 15 min and then preserved at −80 °C until sectioning as previously described.^52^ Prior to the IF assay preparation, multiple scaffold sections of ∼1 mm were generated. Briefly, the preparation included overnight fixation in 4% w/v paraformaldehyde, then 2 h permeabilisation in 0.1% Triton X-100 (Sigma Aldrich, UK) at room temperature followed by 4 h blocking in 10% of donkey serum (Abcam, UK), overnight primary antibody staining, 6 h secondary antibody staining and overnight counterstaining all at 4 °C. Cells and scaffold structure were evaluated with a Leica SP5 inverted confocal microscope and processed with Leica LAS AF software (Leica, Milton Keynes, UK). Each step employed solvents of either 1% w/v bovine serum albumin (Sigma-Aldrich, UK), 0.5% v/v Tween-20 (Promega, UK) and 0.01% w/v NaN_3_ in PBS, or only 0.01% w/v NaN_3_ in PBS after secondary antibody staining. Each step was separated by at least two washes with above solvent buffer. Primary antibodies and isotype controls are summarised in Table 1. Secondary antibodies consisted of: donkey anti rat Alexa Fluor 488 (AF 488), donkey anti mouse Alexa Fluor 555 (AF 555) and donkey anti rabbit 647 (AF 647; all Fisher Scientific, UK) at 1 : 500 dilution. Counterstain consisted of: 5 μg mL^−1^ DAPI (Fisher Scientific, UK).

**Table 1:** Primary and isotype antibody concentrations for the immunofluorescence (IF) assay^a^

	Primary	Isotype
Fig. 4a	11.17 μg mL^−1^ rat anti human Ki-67	11.17 μg mL^−1^ rat IgG1^b^
	16.67 μg mL^−1^ mouse anti human HIF-1α	16.67 μg mL^−1^ mouse IgG2b
	8.34 μg mL^−1^ rabbit anti human collagen-I	8.34 μg mL^−1^ rabbit IgG
Fig. 4b and c	8.00 μg mL^−1^ rabbit anti human LC3 A/B	8.00 μg mL^−1^ rabbit IgG
	16.67 μg mL^−1^ mouse anti human HIF-1α	16.67 μg mL^−1^ mouse IgG2b
Fig. 6	16.67 μg mL^−1^ mouse anti human HIF-1α	16.67 μg mL^−1^ mouse IgG2b
	8.34 μg mL^−1^ rabbit anti human collagen-I	8.34 μg mL^−1^ rabbit IgG
Fig. 7	11.17 μg mL^−1^ rat anti human Ki-67	11.17 μg mL^−1^ rat IgG1^b^
	16.67 μg mL^−1^ mouse anti human HIF-1α	16.67 μg mL^−1^ mouse IgG2b
	8.34 μg mL^−1^ rabbit anti human collagen-I	8.34 μg mL^−1^ rabbit IgG

a All products were obtained from Abcam, UK.

b IgG: immunoglobulin G.

### 2.7. Cell viability confocal imaging

As described in section 2.6, scaffolds were collected at day 29, snap frozen in liquid nitrogen for 15 min and then preserved at −80 °C. After sectioning, each scaffold section was incubated with Calcein-AM (2 μM; Life Technologies, Paisley, UK) and ethidium homodimer-1 (4 μM; Life Technologies) in culture medium for 1 h at 37 °C. The presence of live (green) and dead (red) cells was immediately evaluated with Leica SP5 inverted confocal microscope and processed with Leica LAS AF software (Leica, Milton Keynes, UK).

### 2.8. Confocal laser scanning microscopy (CLSM) imaging

Immunofluorescent samples were imaged on a Leica SP5 inverted confocal microscope and processed with Leica LAS AF software (Leica, Milton Keynes, UK) using 405, 453, 488, 543, and 633 nm lasers and filters for DAPI, reflectance, AF488, AF555, and AF647 stains for 2 sequential scans. Confocal images were captured using a 10× dry objective, with a 512 × 512 pixel resolution and 5 μm *Z*-stack distance, as previously described.^52^ Those image acquisition settings were identical for the positive and the corresponding isotype control. Multiple replicates from multiple scaffold areas where imaged and representative images are presented.

### 2.9. Statistical analysis

Statistical analyses were performed from *N* = 3 independent cultures where *n* = 2–3 replicate scaffold measurements per culture were averaged. Error bars represent standard deviation. Analysis of variance (One-way or Two-way ANOVA) and unpaired, 2-tailed Student's *t*-test was performed using GraphPad Prism® software with a *p*-value threshold 0.05 to evaluate whether there was a statistical difference between the experimental conditions under study.

## 3. Results

### 3.1. Long term cultivation of pancreatic cell lines in the PU scaffolding system

**Fig. 2:**
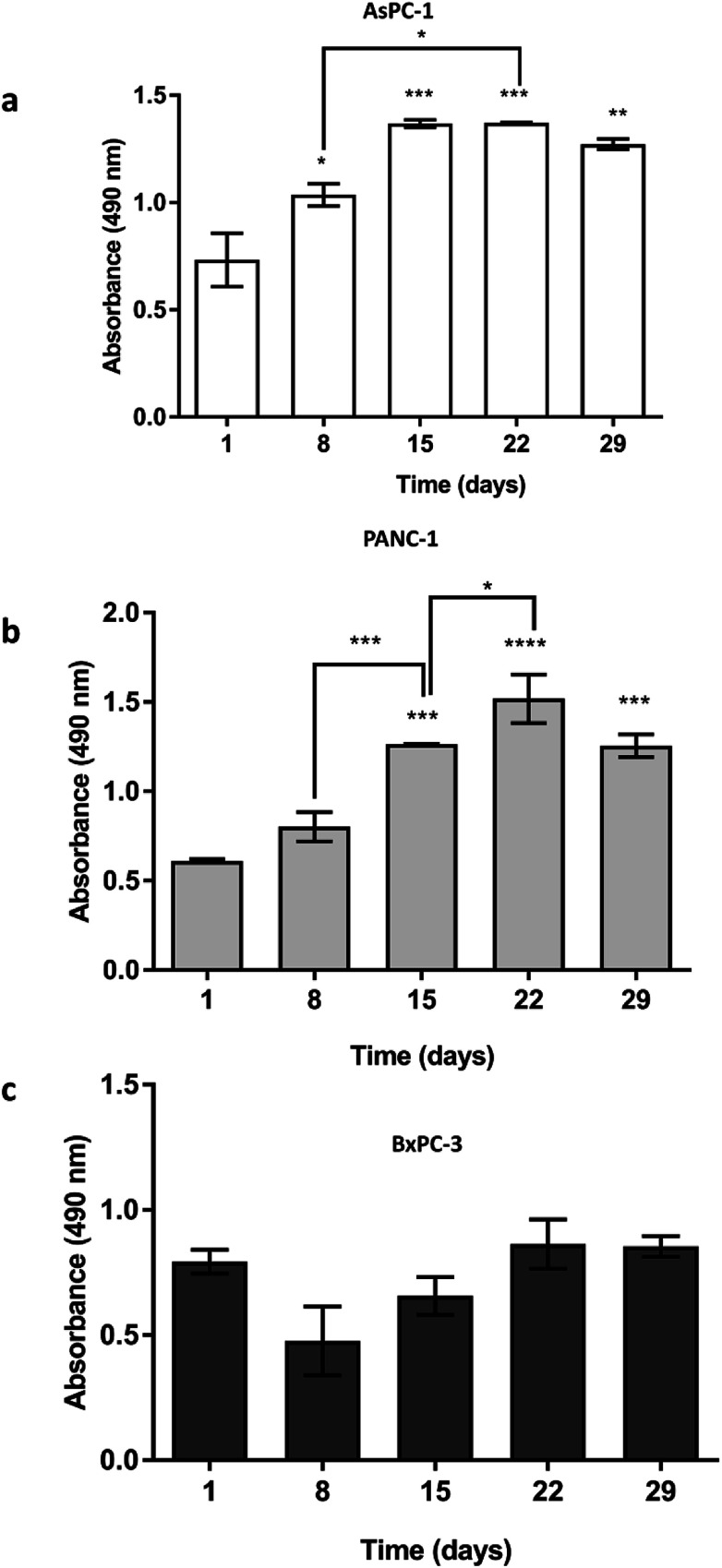
Growth of (a) AsPC-1, (b) PANC-1 and (c) BxPC-3 pancreatic cancer cell lines in uncoated PU scaffolds for 29 days. Data are presented as mean ± S.D (*N* = 3, *n* = 3). Statistical differences for the cell growth are marked by asterisks (*p < 0.05; **p < 0.01; ***p < 0.001; ****p < 0.0001). *N* = number of independent experiments; *n* = number replicates.

AsPC-1, PANC-1 and BxPC-3 pancreatic cancer cell lines were seeded (5 × 10^5^ cells) in uncoated PU scaffolds and cell viability/proliferation was monitored for 29 days. As shown in Fig. 2, high viability was maintained for all the cell lines in the 3D scaffolds. Furthermore, a significant increase in the proliferation took place, for the AsPC-1 and PANC-1 cell lines (Fig. 2a and b). BxPC-3, did not present any significant increase in absorbance indicating a low level of growth rate (Fig. 2c), which is consistent with previously reported 2D studies.^72^

### 3.2. *In situ* cellular characterisation in the PU scaffolding system

#### Cell self-organisation in the 3D cultures

**Fig. 3:**
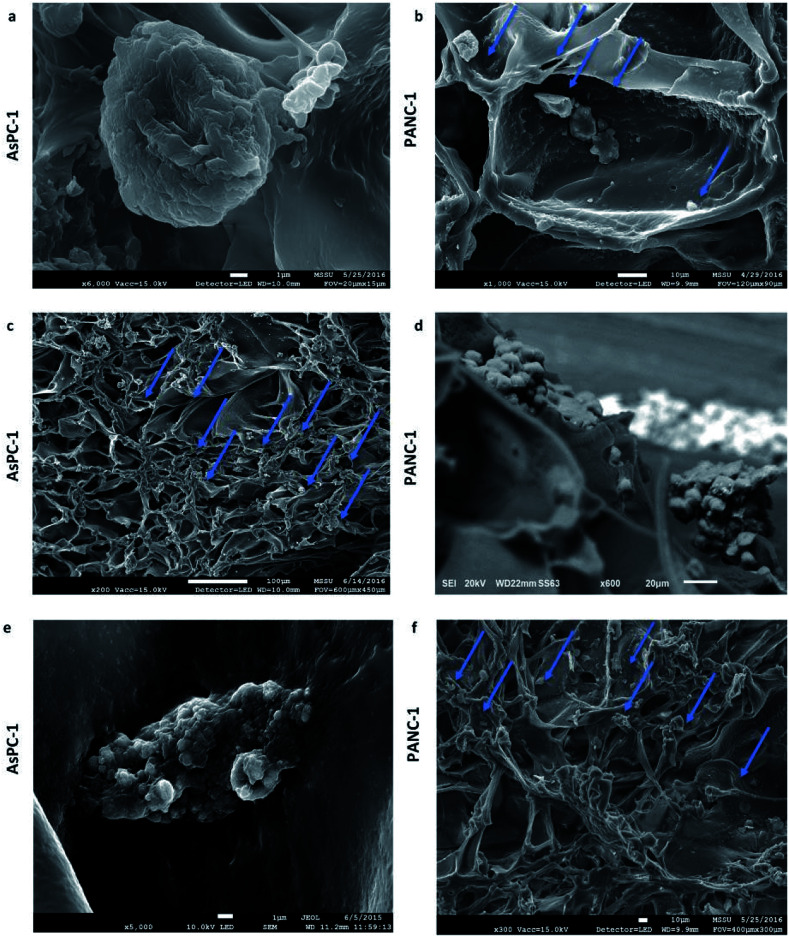
Scanning electron microscopy images of AsPC-1 and PANC-1 cells in sections of the uncoated (UN) PU scaffolds at day 1 (a and b) and day 29 (c–f) of culture.

Scanning electron microscopy (SEM) micrographs verified the ability of the uncoated PU scaffolds to support cellular self-organisation within scaffold pores. As can be seen in Fig. 3 pancreatic cancer cells were distributed as single cells at the beginning of the culturing period (Fig. 3a and b), and formed dense masses at the culture endpoint (Fig. 3c–f).

#### Long term proliferation and maintenance of dense cellular masses in the 3D cultures

**Fig. 4:**
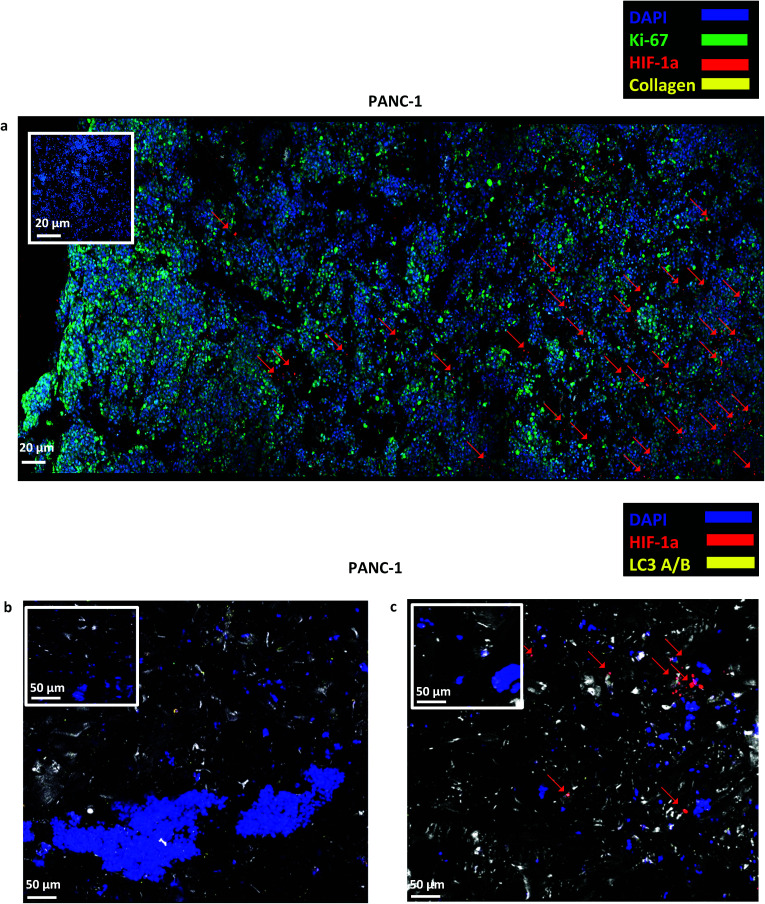
(a) Representative immunofluorescence CLSM images of Ki-67, HIF-1α and collagen-I distribution in sections of uncoated PANC-1 PU scaffolds at day 29 of culture. (b and c) Representative immunofluorescence CLSM of HIF-1α and LC3-A/B distribution in uncoated PANC-1 PU scaffolds at day 1 (b) and day 29 (c) of culture. For each image the corresponding isotype control was generated.

As described in section 2.6, *in situ* immunofluorescent staining of scaffold sections took place. Only scaffolds with PANC-1 cells were used for this purpose, as these cells are considered more aggressive than AsPC-1 and BxPC-3.^73^ A high number of cells (blue) was present and homogeneously distributed throughout the scaffold section from which a significant amount was proliferative (Ki-67 positive) on day 29 of culture (Fig. 4a). Furthermore, the cells that were closer to the edges of the scaffold have not experienced oxidative stress (HIF-1α negative) and a region of hypoxic cells (see red arrows in Fig. 4a and c) was present at the centre, which confirms that the highly porous scaffolding system provided sufficient oxygen for long term cell growth. Collagen-I secretion was not detected in the uncoated PU scaffolds. In order to further investigate the possible presence of nutrient gradients in the centre of the scaffolding system, cells were stained with LC3-A/B antibody (yellow), the autophagic marker that is mainly overexpressed as a response to nutrient deprivation (metabolic stress).^8,74^ As indicated in Fig. 4b and c, the cells were LC3 negative both at the beginning and at the end of the culturing period.

### 3.3. Engineering the PU scaffolding system to recapitulate ECM features

#### The ECM mimicry enhanced pancreatic cancer cell growth in the scaffolds

**Fig. 5:**
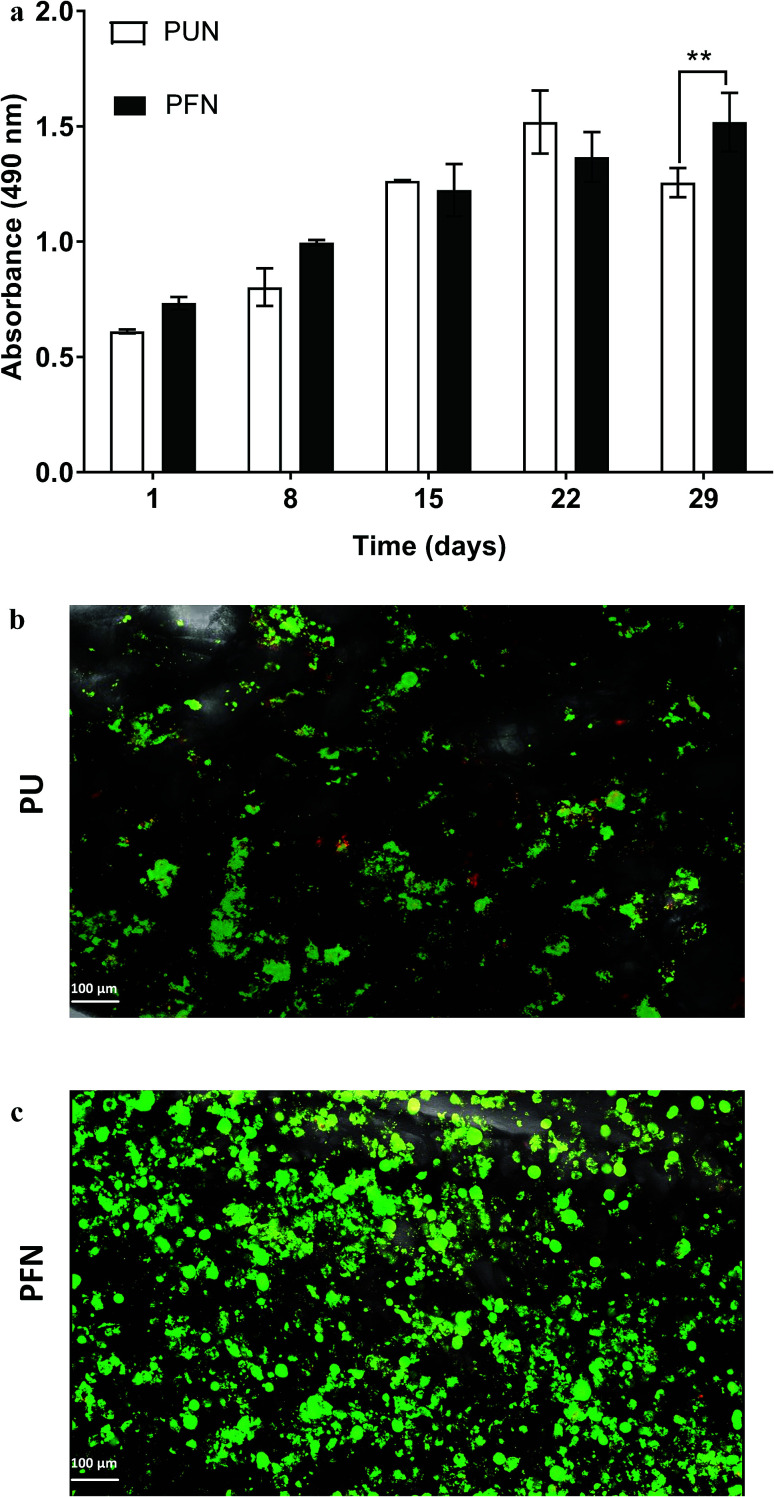
(a) Growth of PANC-1 cells in uncoated (PUN) and FN coated (PFN) PU scaffolds for 29 days. Data are presented as mean ± S.D. (*N* = 2, *n* = 3). Statistical differences are marked by asterisks (*p < 0.05; **p < 0.01). *N* = number of independent experiments; *n* = number replicates. (b and c) Visualization of PANC-1 cells in uncoated (PUN) and FN coated (PFN) scaffolds respectively with fluorescence Live (green, Calcein AM) and Dead (red, Ethidium homodimer-1) viability assay at day 29 of culture.

The PU scaffolds were coated with fibronectin, one of the key proteins in the pancreatic cancer ECM network,^75^ to enhance the cell adhesion on the polymeric matrix. Fig. 5a shows the growth kinetics of PANC-1 cells seeded and cultured in uncoated (PUN) and FN coated (PFN) PU scaffolds. The FN coated scaffolds significantly promoted the cellular proliferation (*P* < 0.01) as compared to the uncoated scaffolds at day 29 of culture (Fig. 5a). This is further confirmed with the *in situ* live/dead staining results on the 29^th^ day of culture that revealed a greater number of live cells masses within the FN coated scaffolds as compared to the uncoated ones (Fig. 5b and c). Nevertheless, all the PU scaffolds (uncoated/FN coated) presented high viability validating the biocompatibility of this scaffolding system for pancreatic cancer cells (Fig. 5b and c).

#### *In situ* fluorescence imaging of FN coated 3D scaffolds for determination of the spatial distribution of cells biomarkers with CLSM

**Fig. 6:**
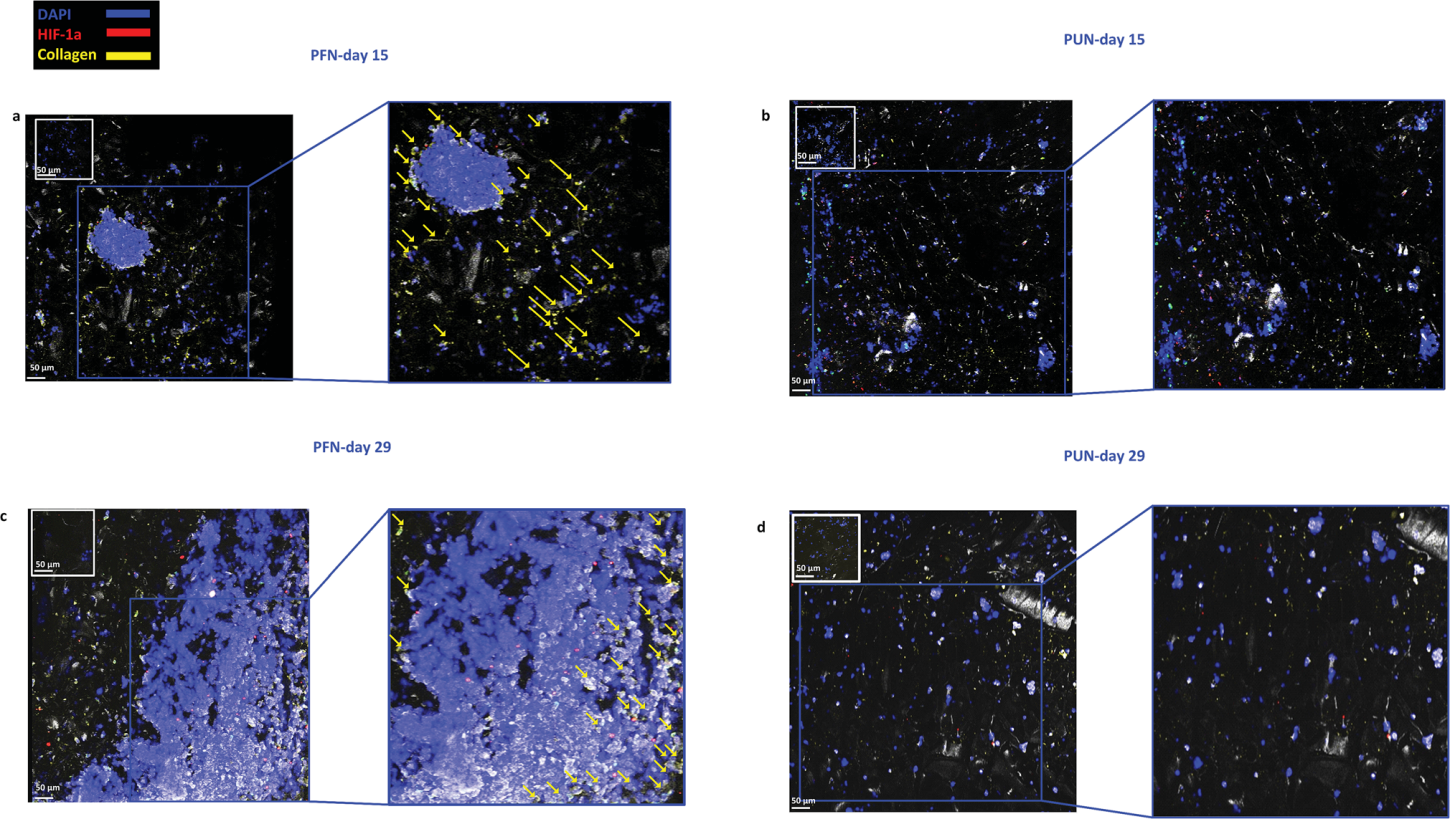
Representative immunofluorescence CLSM images of HIF-1α and collagen-I distribution in FN coated (a and c) and uncoated (b and d) PANC-1 scaffolds at day 15 and day 29 of culture. For each image the corresponding isotype control was generated.

As previously described, the coated scaffolds were sectioned and appropriately stained to determine the cell spatial growth, organisation, ECM production and environmental stress response. CLSM imaging revealed that cellular growth and spatial organisation is influenced by the coating of the scaffolds with FN (Fig. 6). Cells within the FN coated scaffolds (PFN) formed dense masses at day 15 (Fig. 6a) which expanded through day 29 (Fig. 6c). In contrast, cells within uncoated scaffolds (UN) formed smaller masses (Fig. 6b and d). Collagen-I is overexpressed in pancreatic cancer tumours *in vivo* and therefore it was selected as an ECM marker.^76^ The scaffold IF staining demonstrated that collagen-I detection varied between FN coated *versus* uncoated ones. A greater number of cells were imaged secreting collagen-I in FN coated scaffolds (indicated with yellow arrows in Fig. 6a and c). However, no collagen-I production was detected in uncoated scaffolds (Fig. 6b and d).

**Fig. 7:**
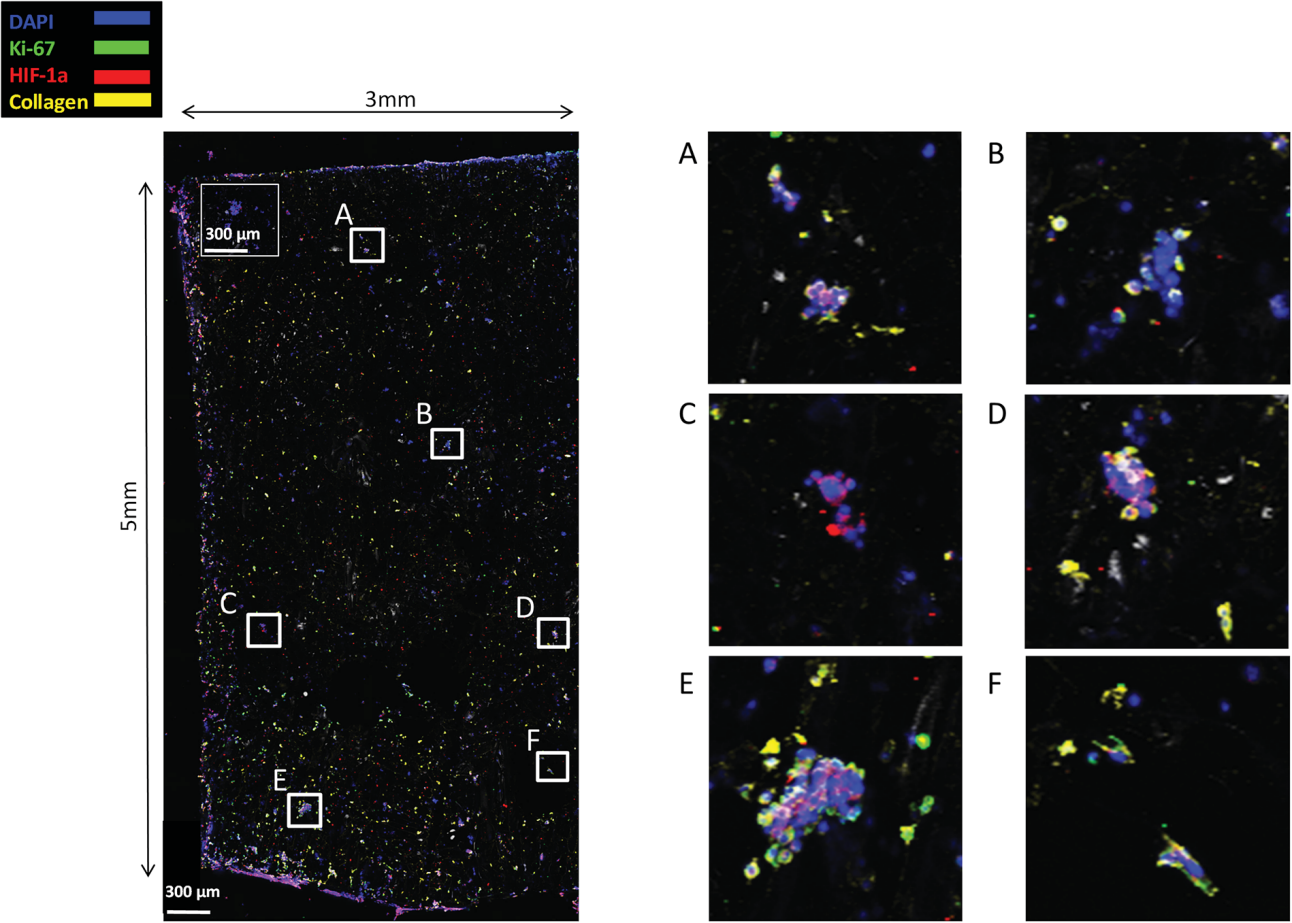
CLSM image of Ki-67, HIF-1α and collagen-I distribution in a wide PANC-1 FN coated scaffold area at day 29 of culture. For each image the corresponding isotype control was generated.

Finally, staining and imaging a wide scaffold area at the endpoint of the culture provides a map of the cellular distribution, the ECM production and the oxidative stress (HIF-1α) biomarker expression within the scaffold. Spatial variations in collagen-I, HIF-1α and Ki-67 expression as well as cell self-organisation are presented in Fig. 7. Different densities of cell masses, most of them maintaining their proliferative properties (Ki-67 positive), were formed within the PU matrix. The cell masses were surrounded by collagen-I molecules and expressed locally oxidative stress gradients (HIF-1α) (Fig. 7).

## 4. Discussion

Overall, in this work, a 3D highly porous PU scaffold coated with fibronectin (FN) was developed and assessed as an *in vitro* platform for pancreatic cancer studies. The developed scaffolding system was able to support the long term cellular growth and proliferation (Fig. 2, 4a and 5–7), cell self-organisation (Fig. 3 and 5–7) and ECM protein, *i.e.*, collagen I, production of pancreatic cancer cells (Fig. 5 and 6). Furthermore, *in situ* mapping of various environmental stress biomarkers, *i.e.*, oxygen and nutrient stress, revealed the formation of some oxygen/oxidative stress gradients, *i.e.*, formation of some hypoxic areas, but no nutrient/starvation stress gradients within the scaffold (Fig. 4, 6 and 7). This is crucial, as the accurate topological identification of stress biomarkers' signals can lead to a better understanding of the 3D tumour response and/or resistance to treatment, *e.g.*, areas of higher or lower resistance.^77,78^ Furthermore, the fact that the environmental gradients are not extreme is of importance as most currently available 3D systems for pancreatic cancer induce artificially high oxygen gradients that lead to a large non-proliferative centre, which is not always present *in vivo*.^32^ To our knowledge, this is the first study that reports the long term growth and evolution of pancreatic cancer cells in a highly porous ECM coated PU scaffolding system. Some previous studies have explored pancreatic cancer cell evolution in 3D polymeric systems, however it is the first time that the PU is used as scaffolding material to generate a highly porous sponge-like matrix which, to the authors' knowledge, resulted to the longest reported cell proliferation period of pancreatic tumour cells, along the study of Chiellini *et al.* Most pancreatic cancer studies in 3D systems to date report an *in vitro* culture period of 7–14 days.^46,49–51^ Long term cultivation is important as it could enable the conduction of fractionated treatment followed by long term post-treatment observations.

### Evolution of pancreatic cancer cells in the highly porous PU scaffold

The developed highly porous PU scaffolding system supported the long term cultivation and proliferation of 3 different PDAC cell lines, *i.e.*, AsPC-1, PANC-1, BxPC-3, for 29 days (Fig. 2). It is noteworthy that the pancreatic cancer cell lines maintained their intrinsic kinetics and typical morphological characteristics within the polymeric matrices (Fig. 2 and 3). AsPC-1 and PANC-1 cells had similar growth rates in the scaffolding system, while BxPC-3 cell line had a slower growth evolution, which is in alignment with the reported duplication times of these cell lines.^72^ In terms of morphological characteristics within the 3D scaffold, as verified by SEM imaging, the AsPC-1 cells retained their foci-like epithelial shape (Fig. 3a),^79,80^ while PANC-1 cells had an elongated epithelial morphology (Fig. 3b).^81,82^ This scaffolding system allowed large population of cells to grow, the majority of which were positive proliferative (Ki-67 positive) on day 29 (Fig. 4a). Similar images of cellular masses during PDAC evolution in a rat model were reported by Ignat *et al.*^83^ Likewise, another study for PDAC detection demonstrated dense cell masses in the tumour area of human pancreatic cancer xenografts.^84^ This suggests that this scaffolding system provides realistic *in vivo* recapitulation of the pancreatic tumour self-organisation facilitating high cellular densities and cell–cell interactions within the 3D structure.

The lack of nutrient gradients despite the high cell densities on day 29 of PANC-1 scaffold culture was verified since the LC3 autophagic marker (Fig. 4b and c), a marker which is mainly upregulated under starvation conditions, was not expressed.^85^ Nevertheless, local hypoxic areas were present mainly where dense overpopulated masses exist, mimicking the low oxygen levels that naturally occur within the pancreatic tumour (Fig. 4a). Similar results of HIF-1α expression in patient derived samples were demonstrated by Salnikov *et al.*^86^ Other studies have also reported the mimicry of hypoxic areas *in vitro*, leading however to extreme oxygen gradients which lead to high apoptotic regions which are not always reported *in vivo*.^87–89^

### Evolution of pancreatic cancer cells in FN coated highly porous PU scaffolds

FN is a major ECM constituent of PDAC that mediates cell adhesion. We therefore, modified further the PU scaffolds with FN, to promote the cell adhesion, but also to provide partially the ECM mimicry to the cells. Cellular viability/proliferation in FN coated scaffolds was significantly higher as compared to the uncoated scaffolds at day 29 of culture (Fig. 5a). However, the difference in cell growth between the uncoated and FN coated scaffolds was noticeably smaller than other reported biological systems in PU scaffolds,^55^ showing that pancreatic cancer cell lines grow significantly well in the PU scaffolds even without ECM coating support. Similarly to our findings, Raza *et al.* reported the effect of FN derived peptides on PANC-1 growth in 3D PEG-based hydrogels and showed that PANC-1 cells exhibited higher proliferation at the end of the culture in the FN enriched hydrogels compared to the FN-free gels.^48^ Additionally, Miyamoto *et al.* reported that pancreatic cancer cell lines growth was enhanced on FN coated 2D monolayers compared to the controls (uncoated surfaces) at the end of the culture.^90^ From the above studies in both 2D and 3D systems it is evident that FN stimuli promotes cell growth *in vitro*. Further to the pancreatic cancer cell growth/proliferation we observed that the FN coating affected the cellular spatial self-organisation in the PU matrix. More specifically, the cells formed larger masses in the FN coated scaffolds as compared to uncoated ones where they formed smaller masses (Fig. 6). We have previously reported this formation of PANC-1 cellular masses in carbon nanotube based films.^80^ FN is considered to interact with the cell surface integrin receptors, which may result in enhancing the cell–cell interactions and consequently leading to cell clustering.^75,91^ This evidence is further supported by the group of Da Rocha-Azevedo *et al.*, who showed that the cell contraction in clustering requires the presence of FN.^92^ Furthermore, the secretion of collagen-I was monitored, as collagen-I is an important feature of PDAC microenvironment which is highly secreted within the densely-packed pancreatic tumour stroma, as proven by patient and animal studies.^76,93,94^ We observed a significant collagen-I secretion in the FN coated scaffolds (Fig. 6a, c and 7) and no collagen-I secretion in uncoated scaffolds (Fig. 4a, 6b and d). Expression of high collagen levels from the pancreatic cancer cells in multilayer FN–gelatin nanofilms was also reported by Matsusaki *et al.*^95^

Finally, sectioning and imaging of a wide scaffold area of the FN coated scaffold (essentially ‘zooming-out’ the FN scaffold) enabled the biomarker mapping of a tissue-scale area, therefore, providing insightful information about on formed cell heterogeneity. It should be stated that, to best of our knowledge, this is the first reported image of this size (∼3 × 5 × 1 mm^3^), as most CSLM images of both *in vivo* and *in vitro* systems are within the range of microns.^46,49,83,84,86^ As can be seen in Fig. 7, the majority of the cell population that existed in the scaffold is proliferative (Ki-67) positive on day 29 of culture, revealing the long term maintenance of the cell culture within the 3D matrix. At this point it should be mentioned that there are very few 3D systems studying the pancreatic cancer cell Ki-67 expression and none after 15 days of culture, as the centre of such 3D models usually contains arrested proliferation and accumulation of apoptotic due to oxidative stress regions.^41,43,89^ The pore size and interconnection of this scaffolding system allows the cells to proliferate throughout the matrix area, with local hypoxic areas (HIF-1α positive) being heterogeneously distributed throughout the scaffold area (Fig. 7). This is of great importance, since hypoxia is a hallmark of all the solid tumours and due to the structural and functional abnormalities of tumour microcirculation spatial and temporal heterogeneity in the perfusion is caused.^96,97^ Heterogeneous HIF-1α gradient accumulation in pancreatic tumours has also been reported for patient derived tissues.^86,98^ Additionally, collagen-I was heterogeneously expressed by the cells within the FN coated scaffolds (Fig. 7).

## 5. Conclusions

Overall, in this study we developed a 3D highly porous PU scaffolding system coated with FN which shows great potential as a model for pancreatic cancer studies. The reported 3D model was able to (i) support long term growth and proliferation of pancreatic cancer cells for up to a month, (ii) allow formation of dense cellular masses, (iii) enhance the collagen-I production from the pancreatic cells and (iv) induce environmental gradients (hypoxic regions) which were not acute and therefore did not arrest cell proliferation. Furthermore, *in situ* mapping of a wide scaffold area revealed a high level of heterogeneity with respect to biomarker spatial distribution. Similar trends have been reported *in vivo*, indicating the great potential of the developed PU scaffolding system for pancreatic cancer studies. Future work will focus on (i) introducing perfusion in the system for vascularisation mimicry as well as (ii) co-culturing of pancreatic cancer cells with stromal cells in order to simulate even more accurately the pancreatic cancer tissue microenvironment.

## Conflicts of interest

There are no conflicts to declare.

## Supplementary Material
